# Oral antibiotics perturbation on gut microbiota after prostate biopsy

**DOI:** 10.3389/fcimb.2022.959903

**Published:** 2022-08-16

**Authors:** Joseph Kai Man Li, Lynn Lin Wang, Becky Su Yan Lau, Ryan Tsz Hei Tse, Carol Ka Lo Cheng, Steven Chi Ho Leung, Christine Yim Ping Wong, Stephen Kwok Wing Tsui, Jeremy Yuen Chun Teoh, Peter Ka Fung Chiu, Chi Fai Ng

**Affiliations:** ^1^ S.H. Ho Urology Centre, Department of Surgery, Prince of Wales Hospital, The Chinese University of Hong Kong, Hong Kong, Hong Kong SAR, China; ^2^ Metabolic Disease Research Centre, Zhengzhou Central Hospital Affiliated to Zhengzhou University, Zhengzhou, China; ^3^ School of Biomedical Science, The Chinese University of Hong Kong, Hong Kong, Hong Kong SAR, China

**Keywords:** antibiotics, gut microbiota, prostate biopsy, bacteria, fecal microbiota

## Abstract

**Introduction:**

The use of antibiotics may induce the changes in gut microbiota. Previous studies have shown conflicting results on whether the changed gut microbiota by antibiotics can be recovered. Our study aims to investigate whether the gut microbiota could be recovered after a single dose of oral co-amoxiclav before transrectal ultrasound-guided transperineal prostate biopsy (TPPBx) in 5 weeks’ time.

**Methods:**

Fifteen patients with elevated serum prostate-specific antigen (PSA) were recruited to provide pre-antibiotic and post-antibiotic fecal samples. The V4 region of 16S rRNA was sequenced. Analysis was performed by QIIME2. Alpha- and beta-diversities were analyzed, as well as the differential enrichment by Linear discriminant analysis Effect Size (LEfSe) analysis.

**Results:**

Both the alpha- and beta-diversities of the pre- and post-antibiotic fecal samples were significantly different. Genera that are associated with alleviation of inflammation were enriched in the pre-antibiotic fecal samples, while the inflammation-associated genera were more enriched in the post-antibiotic fecal samples.

**Conclusion:**

A single dose of oral co-amoxiclav before TPPBx could have led to a change of gut microbiota that cannot be recovered in 5 weeks' time. Microbiome studies on prostate cancer patients should be cautioned on the use of post-prostate biopsy fecal sampling. Further studies should be conducted for the impact on gut microbiome for TPPBx alone.

## Introduction

Normal gut microbiota accommodates approximately 1,000 different bacteria species with more than 7,000 different strains. The term normal bacteria community is defined as commensal bacteria coexisting in the gut of healthy individuals (Gevers et al., 2012).

The gut microbiome profile is built from the difference in diet, geography, environmental exposures, and even genetics. Age, sex, diet, drugs, probiotics, and prebiotics all contribute to the creation of gut microbiota, which, in turn, continues to change throughout its life span (Hollister et al., 2014; Jandhyala et al., 2015; Tuddenham and Sears, 2015).

Gut is an organ that is home to a great diversity of bacteria species, where phyla Firmicutes and Bacteroidetes share the majority of core microbiome in healthy human, followed by Verrucomicrobia and Actinobacteria (Hollister et al., 2014).

The microorganisms in the gut play an important role in maintaining essential functions of healthy people, such as vitamin synthesis, nutrient metabolism, modulating the immune response, and protecting the host against pathogen infection (Lloyd-Price et al., 2016). Once there is an imbalance of gut bacteria, microbiome dysbiosis would be induced, systematic immune response would be altered, and the ability to fight infection would be suppressed (Denny et al., 2016; Langdon et al., 2016). Other health problems like depression, anxiety or psychosis, diabetes, and obesity have also been associated with dysbiosis (Cox and Blaser, 2015; Lurie et al., 2015).

There is increasing evidence that the gut microbiome is important to study the impact of disease development or even treatment response (Tinsley and Cook, 2020).

Antibiotics exposure is considered as one of the major risks to induce gut microbiota perturbation and causes dysbiosis. However, different studies have reported the gut microbiome recovery after antibiotics exposure. MacPherson et al. described that the recovery of gut bacteria microbiota could be as short as 1 week after cessation of 7 days of co-amoxiclav on a healthy adult with a mean age of 34 years (MacPherson et al., 2018). However, another study on 18 subjects with a mean age of 25 with 5 days co-amoxiclav showed the failure of microflora resilience even 2 months after cessation of antibiotics (Mangin et al., 2012).

Transrectal ultrasound-guided transperineal prostate biopsy (TPPBx) has largely replaced transrectal prostate biopsy (TRUS+Bx) as the procedure of choice by patients with high prostate-specific antigen (PSA) due to the high infection rates of TRUS+Bx. In addition, the overall infection rate after TPPBx was approximately 1% (Chiu et al., 2021; Gunzel et al., 2021; Castellani et al., 2022; Jacewicz et al., 2022). However, the use of prophylactic antibiotics is still in place. In the present study, we assess whether the gut microbiota could be affected by a single dose of oral co-amoxiclav with the use of 16s v4 amplicon sequencing from stool samples of patients who underwent TPPBx.

## Methods

### Study design

This was a longitudinal study that has obtained institutional ethics approval and that was conducted according to the Declaration of Helsinki. The study was registered at the ClinicalTrials.gov with identifier number NCT04687709. All patients were recruited from the urology clinics at the Prince of Wales Hospital, Hong Kong SAR. After written informed consent was obtained, a sterile container was provided to the patient for sampling the fecal specimens at their convenience, and the specimen was delivered to the laboratory within 2 h. The samples were then immediately stored at a -80°C freezer until DNA extraction. The study included men suspected of having prostate cancer undergoing prostate biopsy. Men with PSA up to 20 ng/ml were included. The mean age of the study cohort is 66.9 years, and the mean PSA is 9.4. Men with known prostate cancer and prior usage of antibiotics were excluded. Before biopsy, fecal samples were collected as the pre-antibiotics samples. A single dose of 1 g of co-amoxiclav was then given to patients and biopsy was performed. The choice of antibiotics is based on local antibiogram. Five weeks after biopsy, another fecal sample was collected as the post-antibiotics sample.

### Library construction and 16S rRNA sequencing

Microbial DNA was extracted using the QIAamp PowerFecal Pro DNA Kit as previously described (Lim et al., 2020). The V4 regions of the 16S rRNA gene were amplified using specific primers 515F-806R, with 515F forward primer: 5′GTGCCAGC MGCCGCGGTAA3′ and 806R reverse primer: 5′GGACT ACHVGGGTWTCTAAT3′, with the combinatorial dual barcodes. All PCR reactions were performed with Phusion^®^ High-Fidelity PCR Master Mix (New England Biolabs) according to the manufacturer’s instruction. During the library preparation, combinatorial dual indexing was applied. The libraries were constructed by using the NEBNext^®^ UltraTM DNA Library Prep Kit and then paired-end sequencing was performed by Illumina NovaSeq 6000 Sequencing System.

### Data processing using QIIME2

The raw reads were utilized to obtain clean reads after demultiplexing based on unique barcodes and removing primer sequences. Quality filtering on the clean reads was performed using q2-dada2 plugin (Cisternas, 2016) implemented in QIIME2 (v2019.7) (Bolyen et al., 2019) according to the user manual. The output file of dada2 was a feature table including all amplicon sequence variants (i.e., ASVs table). For alpha-diversity and beta-diversity analyses, the sampling depth of 29,000 was used based on the dada2 feature table summary to retain more sequences per sample while excluding as few samples as possible. All alpha-diversity and beta-diversity indexes were computed using q2-diversity and plotted by the “microeco” package (v0.9.0) (Liu et al., 2021). The principal coordinate analysis (PCoA) plots based on Jaccard and Unweighted distances were displayed using the “microeco” package. For taxonomic annotation, feature sequences were assigned to pretrained naive Bayes classifier trained on the SILVA 138 99% OTUs from 515F/806R region of sequences (Quast et al., 2013). The taxonomic composition bar plot of samples among groups at the phyla level was generated using the “microeco” package. As the microbiota was presented in relative abundance, linear discriminant analysis (LDA) effect size (LEfSe) analysis (Segata et al., 2011) was used to compare the differences in microbiota composition.

### Linear discriminant analysis effect size analysis

The LEfSe analysis was calculated on the Galaxy instance of the Hutlab (https://huttenhower.sph.harvard.edu/galaxy). Galaxy is a web-based scientific analysis platform for analysis of large biomedical datasets, including genomics, proteomics, metabolomics, and imaging (Afgan et al., 2018). Taxonomic biomarkers at the genus level were detected using LEfSe with an LDA score threshold of >2.0, *p* < 0.05.

### Statistical analysis

For alpha-diversity, paired sample Wilcoxon test was adopted to calculate the statistical significance of differences among the groups. PERMANOVA analysis was performed on the Jaccard and Unweighted distance matrices. Differential metabolic pathways between two groups were detected using LEfSe with an LDA score threshold of >2.0, *p* < 0.05. Figures were displayed using the “microeco” package by R (version 4.0 or higher).

## Results

### Demographic data

Fifteen patients who underwent TRUS for prostate biopsy were recruited. The mean age of the study cohort is 66.9 and the mean PSA is 9.4. Eleven patients are benign cases, and four of them have prostate cancer. The demographics and clinical information of patients from whom specimens were taken are summarized in **Table 1**.

**Table 1:** Demographics of patients.

	Patient cohort (%)
**Age**	66.9 ± 7.6^*^
**BMI**	24.4 ± 2.4^*^
**DM**	0 (0%)
**Hyperlipidemia**	7 (46.7%)
**Hypertension**	11 (73.3%)
**PSA**	9.4 ± 3.3^*^
**PCa**	4 (26.6%)
**Gleason scores (3 + 4)**	4 (26.6%)

^*^ Values are represented in mean ± SD.

BMI, body mass index; DM, diabetes mellitus; PSA, prostate-specific antigen; PCa, prostate cancer.

### Taxonomic analysis of gut microbiome

**Figure 1:**
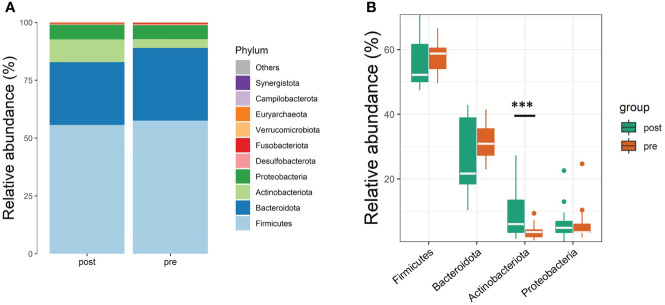
Taxonomic profiles at the phyla level of pre-antibiotics and post-antibiotics groups. **(A)** The relative abundances of the top 10 microbial communities identified at the phyla level between pre-antibiotics and post-antibiotics. **(B)** The differences in the top 4 phyla (Firmicutes, Bacteriodetes, Proteobacteria, and Actinobacteria) between pre-antibiotics and post-antibiotics. ***, p-value < 0.001.

**Figure 2:**
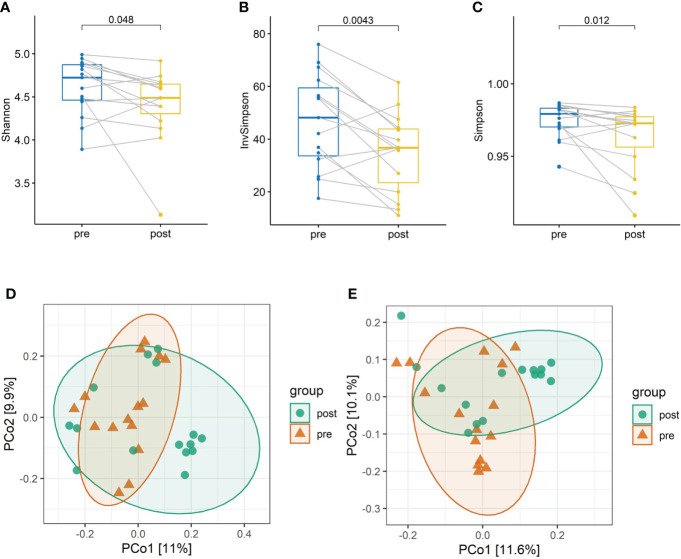
The alpha-diversity and beta-diversity analysis between pre-antibiotics and post-antibiotics groups. Paired comparison of alpha-diversity indexes including **(A)** Shannon, **(B)** Inverse Simpson, and **(C)** Simpson index in the two groups. Microbial communities of samples obtained from the post-antibiotics group display significantly lower diversity than the pre-antibiotics group. Principal coordinates analysis (PCoA) based on Jaccard distance **(D)** and Underweighted distance **(E)** shows dissimilarity of microbial communities between pre-antibiotics and post-antibiotics groups.

The taxonomical composition of the predicted gut microbiota was investigated by sequencing the v4 region of the 16s rRNA gene on the fecal samples. The four major phyla were *Firmicutes*, *Bacteroidota*, *Actinobacteriota*, and *Proteobacteria*. A summary of the taxonomic analysis is presented as a bar chart in **Figure 1A**. *Actinobacteriota* is the only phylum significantly different between pre- and post-antibiotics groups (**Figure 1B**). The microbial diversities of the predicted gut microbiota were compared between the pre-antibiotics and post-antibiotics. All the three alpha-diversity indices were significantly lower in the post-antibiotics samples (Shannon *p* = 0.048, Simpson *p* = 0.012, InvSimpson *p* = 0.0043, **Figures 2A–C**). The beta-diversity was significantly different based on Jaccard and Unweighted dissimilarity (*p* < 0.05, **Figures 2D, E**). This indicates that the composition of the predicted gut microbiota between pre-antibiotics and post-antibiotics groups is significantly different (**Figures 2A–E**).

### Taxonomic differences between groups

**Figure 3:**
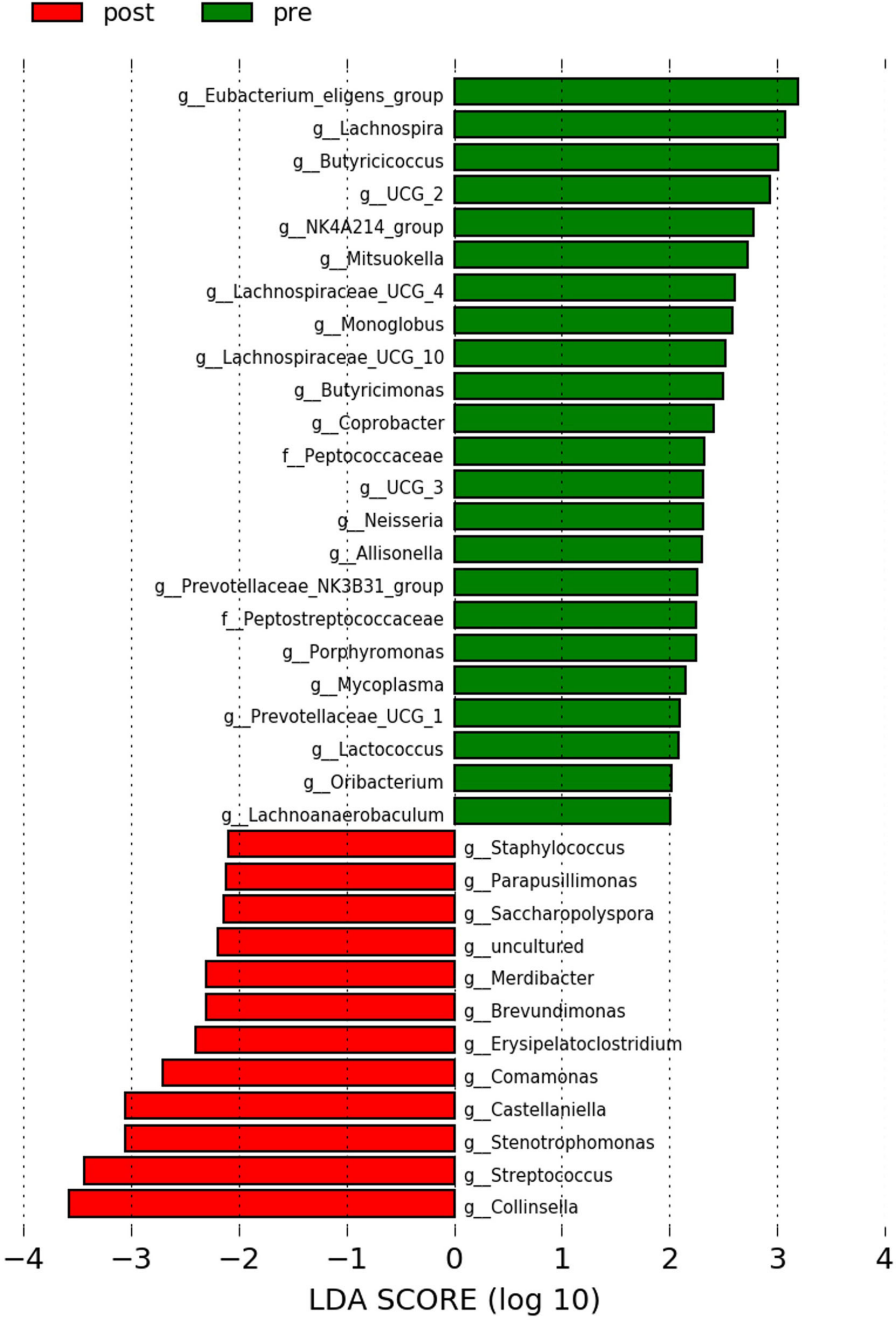
LEfSe analysis between pre-antibiotics and post-antibiotics groups. Histogram of LDA scores is shown as the results of LEfSe analysis for evaluating biomarkers with the statistical difference among groups. Only differentially enriched microbiota with LAD >2.0 and p-value <0.05 were included.

The LEfSe analysis showed that pre-antibiotic samples were more enriched with bacterial genera that are associated with alleviation of inflammation [e.g., *Eubacterium eligens* group, *Lachnospira* (Ramos et al., 2022), and *Butyricicoccus* (Li et al., 2021)], while the post-antibiotic samples were more enriched with inflammation-associated genera [e.g., *Collinsella* (Astbury et al., 2020) and *Streptococcus* (Zhuang et al., 2020)] (**Figure 3**).

## Discussion

Although recently the practice of TPPBx has become more common with its low rate of urinary tract infections and sepsis, the exercise of transrectal biopsy is still ongoing in different clinics and hospitals (Pepe and Aragona, 2013; Pepe and Pennisi, 2022). During transrectal prostate biopsy, prophylactic antibiotics is a common practice in patients to reduce post-biopsy sepsis, and antibacterial prophylaxis is recommended in the European Association of Urology (EAU) guidelines (Morin et al., 2020). Many reports mentioned the drugs’ effect in gut microbiome, especially antibiotics, which can alter the homeostasis of gut normal flora and impose an imbalance of bacteria communities. The reduced gut alpha-diversity refers to the loss of microbial variety or changes in relative abundance of gut bacteria, which is commonly linked to health condition and referred to as dysbiosis (Li et al., 2020; Chen et al., 2021). A growing number of reports demonstrated that even a short course of antibiotics can alter the composition of the bacteria in the gut. According to local bacterial antibiogram, co-amoxiclav is chosen as the prophylactic antibiotic, which works against a wide range of microbes, including both Gram-positive and Gram-negative bacteria (Evans et al., 2022). The effect of a single dose to multiple doses of co-amoxiclav within a week on the gut microbiome has been studied by different groups. The observed gut bacteria recovery ranged from 1 week to a few months (MacPherson et al., 2018; Elvers et al., 2020). However, there was no study on a single dose of co-amoxiclav in the literature.

The alpha**-**diversity is an index for the within-group diversity, which is believed to be healthier if there is greater diversity. The alpha**-**diversity of the gut microbiome is expected to be associated with healthy conditions. The low level of diversity is an indicator of gut microflora imbalance and is always associated with acute and chronic disease, inflammatory diseases, and immune diseases (Pickard et al., 2017; Manor et al., 2020). Our results showed that the alpha-diversity in the post-antibiotic samples was significantly lower than that of the pre-antibiotic samples. It could be implied that the post-antibiotic samples were less healthy. The composition of the pre- and post-antibiotic samples was significantly different as shown by the beta-diversity. These results show that even with a single dose of co-amoxiclav with a 5-week recovery period, the gut microbiota still could not get back to its pre-antibiotic state, and it is significantly less healthy than during the pre-antibiotic state.

From the LEfSe analysis at the genus level, our results showed that the pre-antibiotics samples were healthy gut microbiome-related dominant. The genera *E. eligens* (Mukherjee et al., 2020), *Lachnospira* (Telle-Hansen et al., 2018), *Butyricicoccus* (Eeckhaut et al., 2013), *Mitsuokella* (Ramos et al., 2022), *Butyricimonas* (Garcia-Mantrana et al., 2018), *Coprobacter* (Abis et al., 2019), *Allisonella* (Wang et al., 2021), *Lactococcus* (Shah et al., 2020), and *Oribacterium* (Nardelli et al., 2020) have all been reported to be associated with a healthy gut environment. While the importance of *E. eligens*, *Lachnospira*, and *Mitsuokella* has been reported in short-chain fatty acid production, the regulatory roles of *Butyricicoccus* and *Allisonella* in inflammation have been described. The post-antibiotics enriched bacteria, *Collinsella* and *Streptococcus*, are associated with inflammation (Astbury et al., 2020; Zhuang et al., 2020). Interestingly, our result is in line with previous studies that reported the use of antibiotics to promote inflammation. One of the surprising outcomes of antibiotic therapy has been described previously, where antibiotics could alter the composition of gut microorganisms and increase inflammatory disorders (Knoop et al., 2016). Although our study involved only a single dose of antibiotics, its impact on gut microbiome could not be overlooked.

Previous studies on gut microbiome recovery could be as short as 1 week to as long as 6 months after short-term antibiotics intervention (MacPherson et al., 2018; Elvers et al., 2020), but most of the early studies focused on healthy subjects. On the other hand, there are also publications to suggest the supplement of probiotics to restore the gut microbiome in a shorter time and to reduce the alteration of intestinal bacteria (Hempel et al., 2012; Holloway et al., 2014). In the current study, fecal samples for pre-/post-antibiotics treatment of our patients who are undergoing TPPBx suffered from high PSA. An elevated PSA could be due to prostate cancer, benign prostatic hyperplasia (BPH), or prostatic inflammation. In our study, 26.7% of the cohort were diagnosed with prostate cancer, and the remaining were suffering from BPH. The difference of our results from the literature could be due to the fact that our patients were suffering from a disease and thus were not considered healthy subjects, who were recruited in other studies. Also, the mean age of our patients was 66.9, which was significantly older than the subjects in the literature. There were few studies that demonstrated the importance of age in gut microbiome. In the elderly, antibiotic exposure was linked to microbiota diversity loss (Ng et al., 2019; Ramirez et al., 2020). Recently, the age factor was further demonstrated in a mouse model, where older mice with 10 days of antibiotics could not achieve gut bacteria recovery after 6 months and even at the end of the study (Laubitz et al., 2021). These results revealed that age is a critical factor associated with gut microbiome recovery after antibiotic treatment.

The current study also poses an important time frame for the collection of fecal samples for gut microbiome studies in prostate cancer, especially for studying the pathogenesis of prostate cancer or the use of gut microbiome as a potential biomarker for diagnosis. There has been an exponential growth in the number of studies of microbiome in prostate cancer, but the timing for the stool sampling in patients, particularly before or after prostate biopsy, was not well documented [for example, a recent published article by Kure et al. (2022)]. This could lead to an unnoticed bias to the conclusion.

Our study is not without limitations. Firstly, our cohort did not exhibit a balance between benign and cancer patients; therefore, the microbiome recovery between benign and cancer patients after receiving a single dose of co-amoxiclav could not reach statistical significance. Second, the effect on the gut microbiome may not be solely due to the antibiotic effect, and the effect of TPPBx on the gut microbiome without antibiotics was not previously studied. However, there are recent studies showing that the probability of septic infections after TPPBx without prophylactic antibiotics is low (Basourakos et al., 2022). Further studies could be designed to have patients undergo TPPBx without antibiotics to understand its impact on the gut microbiota. Thirdly, as this is an exploratory study, the number of subjects could not be estimated, and the small sample size could have an impact on the power of the analysis. Nevertheless, our study has the advantage of having a longitudinal study with paired fecal samples. Subjects usually have a core gut microbiota, and by comparing the paired fecal samples, the inter-individual differences are minimized.

In conclusion, our study has demonstrated that a single dose of oral co-amoxiclav before TPPBx could have led to a change in gut microbiota with no recovery up to 5 weeks. Apart from the issue of host health, this also has to be considered during fecal sampling for microbiome studies on prostate cancer. Further studies are required to explore the impact of TPPBx without antibiotics on gut microbiota and to investigate the use of post-TPPBx fecal samples in microbiome studies.

## Data availability statement

The data presented in the study are deposited in the BioProject database repository, accession number PRJNA858302.

## Ethics statement

This study was reviewed and approved by The Joint Chinese University of Hong Kong – New Territories East Cluster Clinical Research Ethics Committee. The patients/participants provided their written informed consent to participate in this study.

## Author contributions

JL, LW, BL, RT, CC, SL, and CW: Writing—original draft preparation, revision, and investigation. JL, LW, BL, RT, CC, SL, and CW: Revision. JL, CW, ST, JT, PC, and CN: Conceptualization. ST, JT, PC, and CN: Supervision. ST, JT, PC, and CN: Validation. All authors contributed to the article and approved the submitted version.

## Conflict of interest

The authors declare that the research was conducted in the absence of any commercial or financial relationships that could be construed as a potential conflict of interest.

## Publisher’s note

All claims expressed in this article are solely those of the authors and do not necessarily represent those of their affiliated organizations, or those of the publisher, the editors and the reviewers. Any product that may be evaluated in this article, or claim that may be made by its manufacturer, is not guaranteed or endorsed by the publisher.
